# Botulinum Neurotoxin Type A Directly Affects Sebocytes and Modulates Oleic Acid-Induced Lipogenesis

**DOI:** 10.3390/toxins14100708

**Published:** 2022-10-15

**Authors:** Karen Brami-Cherrier, Alex Chernavsky, Hui You, Sergei A. Grando, Amy Brideau-Andersen, Birgitte Sondergaard

**Affiliations:** 1 Neurotoxin Research Program, Department of Biological Sciences, Allergan Aesthetics, an AbbVie Company, Irvine, CA 92612, USA; 2 Department of Dermatology, University of California Irvine, Irvine, CA 92617, USA

**Keywords:** BOTOX^®^, onabotulinumtoxinA, binding domain, skin, oiliness, seborrhea, acne, sebum, fibroblast growth factor, 5α-dihydrotestosterone

## Abstract

Excess sebum (seborrhea) results in oily skin and is associated with large pore size and acne. Studies in healthy, seborrheic volunteers have reported that intradermal injection of commercial preparations of botulinum neurotoxin type A (BoNT/A) (onabotulinumtoxinA, abobotulinumtoxinA, and incobotulinumtoxinA) reduced sebum production, and thus, skin oiliness and pore size. The mechanism for these effects has not been fully elucidated; however, several theories involving direct or indirect effects of BoNT/A on neuronal and/or dermal cells (e.g., sebocytes) have been proposed. In the present study, we evaluated the direct effect of native research grade BoNT/A complex, a commercial preparation of BoNT/A (onabotA), and BoNT/A variants on sebocyte lipogenesis using an in vitro sebocyte cell model. We show that picomolar concentrations of BoNT/A (BoNT/A complex: half maximal effective concentration [EC_50_] = 24 pM; BoNT/A 150 kDa: EC_50_ = 34 pM) modulate sebocyte lipogenesis and reduce oleic acid-induced sebocyte differentiation, lipogenesis, and holocrine-like secretion. Comparative studies with the binding domain of BoNT/A, which lacks enzymatic activity, show that this effect is independent of the enzymatic activity of BoNT/A and likely occurs via sebocyte cell surface receptors (e.g., fibroblast growth factor receptors). Overall, these results shed light on the potential mechanism of action and rationale for use of BoNT/A for treatment of sebum-related conditions.

## 1. Introduction

**Figure 1:**
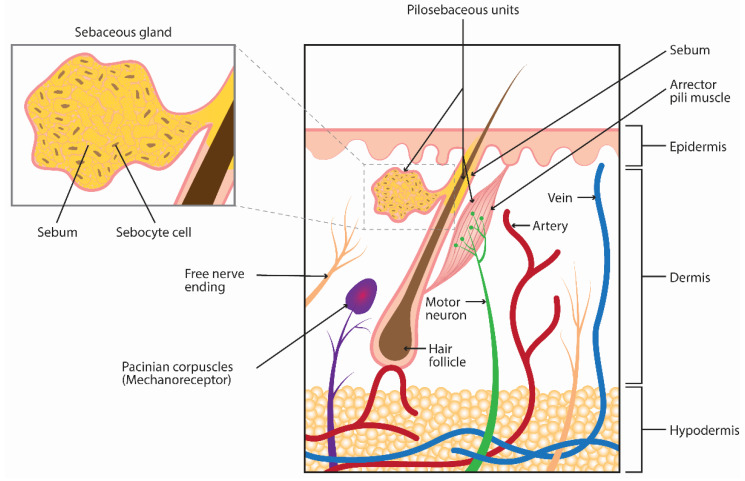
Anatomy of the sebaceous gland.

Oily skin (seborrhea) is a common dermatologic concern resulting from excess production of sebum [1,2,3]. In humans, sebum comprises a mixture of triacylglycerols, diacylglycerols, and free fatty acids (together account for 50–60%), wax esters (20–30%), squalene (10–16%), cholesterol esters (2–4%), and metabolites and debris of sebocytes, or sebum-producing cells [4,5,6]. Sebum functions to maintain skin integrity by reducing water loss and increasing lubrication [7]. Sebum also has anti-bacterial and photoprotective properties [8]. It is produced by sebocytes within sebaceous glands, skin appendages that, with the hair follicle, make up the pilosebaceous unit (Figure 1). Sebocyte stem cells proliferate within the sebaceous gland and then differentiate, accumulating lipids, into mature lipid-containing sebocytes. Once sebocytes become terminally differentiated, they undergo holocrine secretion, where they rupture and release accumulated lipids, or sebum [7,9,10].

Sebum production is influenced by multiple factors [7,11]. Endogenous drivers of sebum production include age and hormones (e.g., estrogens, androgens [including α-dihydrotestosterone], growth hormones, melanocyte-stimulating hormone [α-MSH]) [7,12,13,14,15,16], growth factors (e.g., epidermal growth factor [EGF], fibroblast growth factor 1 [FGF1], insulin-like growth factor 1 [IGF-1]) [5,15,17,18,19], neuropeptides, activity of the hypothalamic-pituitary-adrenal axis (i.e., stress), and acetylcholine (ACh) [20,21,22,23,24]. Environmental factors, such as humidity and UV light, bacterial infection (e.g., *Propionibacterium acnes*), fatty acid consumption, and nicotine use also impact sebum levels [6,7,8]. Although sebum is important in maintaining skin integrity, excess sebum (seborrhea) leads to oily skin that is aesthetically undesirable and associated with acne and large pore size [25,26,27,28]. Current treatment options for seborrhea or oily skin include topical agents (e.g., isotretinoin, spironolactone, other cosmeceuticals) [29], oral contraceptives, and photodynamic and laser therapies [2].

Botulinum Neurotoxin Type A (BoNT/A) is a neurotoxin produced by *Clostridium botulinum*, which causes temporary muscle paralysis by inhibiting ACh release at the neuromuscular junction [30,31]. BoNT/A is a 150 kDa protein comprised of a light chain (LC), which includes a ~50 kDa enzymatic domain, linked by a single disulfide bond and non-covalent interactions to a ~100 kDa heavy chain (HC) containing a ~50 kDa receptor binding (H_C_) domain and a ~50 kDa translocation domain (H_N_). The 150 kDa BoNT/A toxin is associated with several complex proteins including hemagglutinins and a 140 kDa nontoxic nonhemagglutinin (NTNH) protein; together they form the BoNT/A complex [32,33,34]. As part of the well-known classical mechanism of action of BoNT/A, upon motor neuronal intoxication, the receptor binding domain facilitates specific binding and uptake into neurons. Once internalized, endosomal acidification triggers translocation of light chain into the cytosol, where it enzymatically cleaves synaptosomal-associated protein 25-kDa (SNAP-25), which is essential for mediating vesicular fusion and exocytosis. Cleavage of SNAP-25 blocks release of neurotransmitters and neuropeptides, including ACh, from neuronal cells, and is responsible for BoNT/A’s observed pharmacological effects on smooth and skeletal muscles [30,31].

Clinical uses of BoNT/A include multiple therapeutic and aesthetic indications [35,36,37,38]. Clinical studies in healthy, seborrheic individuals have reported that injections of commercial preparations of BoNT/A, including onabotulinumtoxinA (BOTOX^®^, Allergan Aesthetics, an AbbVie company, Irvine, CA, USA), abobotulinumtoxinA (Dysport^®^, Galderma, Lausanne, Switzerland), and incobotulinumtoxinA (Xeomin^®^, Merz Aesthetics, Frankfurt, Germany), reduce sebum production and, thus, oiliness and pore size [39,40,41,42,43,44]. Proposed mechanisms for these effects are of great clinical interest and include a direct effect of BoNT/A on sebocyte glands and indirect effects on arrector pili muscles or free nerve endings proximal to the gland [43,45]. However, the exact mechanism of action for BoNT/A-mediated reductions in sebum production is presently unclear. Models currently used to study sebocytes include primary sebocytes and immortalized sebocyte cell lines, such as SZ95 [46] and SEB-1 (Table 1) [47]. Known stimulators of sebocyte differentiation and lipogenesis in these models include linoleic acid [48], which functions as a substrate for sebum production, dihydrotestosterone (DHT) [14], α-MSH [13], ACh [23,24], calcium chloride (CaCl_2_) [49], IGF-1 [15], and rosiglitazone (an agonist of peroxisome proliferator-activated receptors [PPARs]) [50]. Interestingly, growth factors such as EGF, transforming growth factor alpha (TGF-α), and basic fibroblast growth factor (FGF2) have been shown to promote sebocyte proliferation at the expense of sebocyte differentiation and thereby reduce sebocyte lipogenesis [19,51,52].

In this study, we identified oleic acid (OA) as a potent lipogenic stimulator in sebocytes. OA is a monounsaturated omega-9 fatty acid present in dietary fats such as olive oil. Dietary fatty acids are substrates for sebum and dietary habits have been shown to alter sebum production and composition [6,53]. We tested the effects of OA on a human sebocyte cell line (SEB-1) to establish an in vitro sebocyte model for seborrhea using OA-stimulated lipogenesis. Using this model, we compared the effects of a commercial preparation of BoNT/A (onabotulinumtoxinA; BOTOX^®^), research grade BoNT/A complex and BoNT/A 150 kDa, recombinant wild-type and mutant variants of the BoNT/A binding domain, the BoNT/A complex protein NTNH, and FGFs on naive or OA-stimulated sebocyte lipogenesis. The mechanism of action was further explored using a fibroblast growth factor receptor (FGFR)-specific kinase inhibitor (BGJ398 [Infigratinib]) [54]. We also examined the effects of BoNT/A on OA-stimulated lipogenesis in primary sebocytes from adult donors. We show that BoNT/A directly affects lipogenesis in an in vitro model with a human sebocyte cell line (SEB-1) and in primary sebocytes and show that the effect of BoNT/A is likely due to a non-classical, SNAP-25-independent mechanism of action.

## 2. Results

### 2.1. Oleic Acid-Induced Lipogenesis in Cultured SEB-1 Cells as an In Vitro Model of Oily Skin

**Figure 2:**
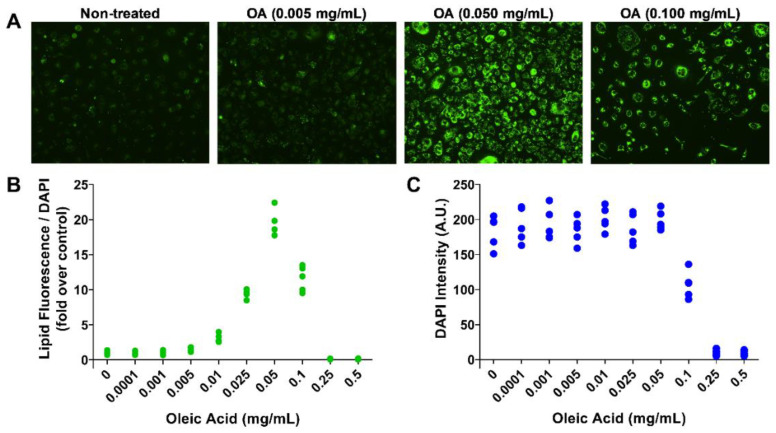
Effect of oleic acid (OA) on sebocyte (SEB-1) lipogenesis and cell viability. (**A**) Microscopic images of representative lipid staining using fluorescent Nile red staining on SEB-1 cells treated with increasing doses of OA (0.005–0.5 mg/mL) for 24 h. (**B**) Dot plot graph of dose response measuring lipid production by lipid droplet fluorescence assay. The ratio of fluorescence over DAPI intensities was normalized with respect to control condition. OA (0.0001–0.5 mg/mL) was tested and measured after 24 h. Each dot represents a replicate (5 replicates per condition). (**C**) Dose–response dot plot showing the cell density variation with increasing doses of OA for 24 h using DAPI staining intensity.

Sebocytes are characterized by their ability to differentiate, accumulate lipid droplets, and eventually rupture and release lipids via holocrine secretion. To generate an in vitro model of oily skin, we used the human sebocyte cell line SEB-1 isolated from the facial skin of a 55-year-old male donor (Table 1) [47]. We tested the ability of 6 known lipogenic stimuli and 1 additional potential stimulus (OA; based on its similarity to linoleic acid) to induce consistent, robust sebocyte lipogenesis and lipid accumulation without holocrine secretion in SEB-1 cells. Doses were informed by available literature for each stimulator (see Section 1) and pilot studies. It should be kept in mind the growth media already contains some of these factors, including FGFs. Treatment time of 1 day was selected since it was sufficient to induce robust lipogenesis and less likely to result in holocrine-like secretion and cell division, both factors that could impact the total amount of lipids per cell. All tested stimuli significantly increased the amount of lipids; DHT and OA led to the highest increase in lipids relative to untreated controls (Figure S1, panel A). Note that since cell viability was not measured, it is possible that treatment with some of these stimulating factors led to holocrine-like secretion and cell death, precluding identification of all effective inducers. This was later observed with higher concentrations of OA (Figure 2). To monitor lipogenesis and cell viability in parallel, DHT and OA were tested again with lipid droplet accumulation normalized to cell number based on DAPI staining (Figure S1, panel B). OA (0.05 mg/mL; ~16-fold over control) was twice as efficacious as DHT (100 µM; ~6-fold over control) at inducing lipogenesis (Figure S1, panel B). Thus, OA was chosen as the lipogenic stimulus for our in vitro model and used in further experiments.

We next examined the dose–response of OA-stimulated lipogenesis in SEB-1 cells. As shown in Figure 2A, the fluorescence intensity, a measure of lipogenesis, increases in cells treated with 0.05 and 0.1 mg/mL OA as compared with non-treated cells. Quantification of fluorescence intensity (Figure 2B) shows a dose-dependent increase in fluorescence intensity, with the highest intensity obtained by treated with 0.05 mg/mL of OA. At higher concentrations (0.1–0.5 mg/mL), the DAPI staining quantification (Figure 2C) reveals a decrease in cell number, due to holocrine-like secretion at higher OA doses. Thus, an OA concentration of 0.05 mg/mL was used as an optimal dose for further experiments with SEB-1 cells.

### 2.2. Effect of onabotA and rH_C_/A on OA-Induced Lipogenesis in SEB-1 Cells

**Figure 3:**
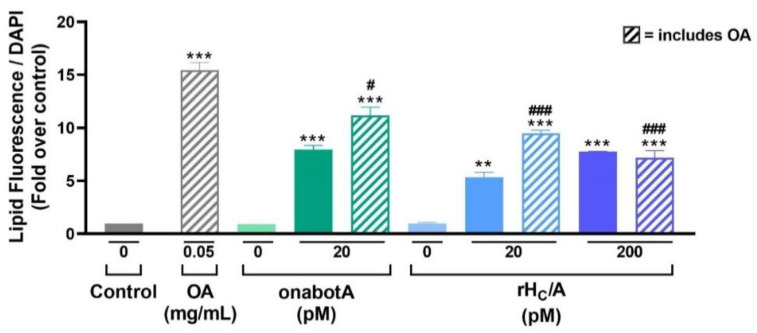
Effect of onabotA and recombinant BoNT/A binding domain (rH_C_/A) on naive and OA-stimulated SEB-1 cells. SEB-1 cells were treated with 0.05 mg/mL of OA alone or in combination with onabotA (100 units in 0.25 mL: ~20 pM) or rH_C_/A (20 or 200 pM), and the amount of intracellular lipids per cell were measured by fluorescence lipid droplet assay and normalized to cell number using DAPI staining. Data shown as fold over control (untreated cells). Although onabotA and rH_C_/A alone induced lipid production, co-treatment with OA decreased their effects on lipid production compared to treatment with OA alone. Data represent means from 5 replicates + SEM. ** *p* < 0.01, *** *p* < 0.001 vs. 0 control; # *p* < 0.05, ### *p* < 0.001 vs. OA (0.05 mg/mL).

Since clinical reports suggest that BoNT/A has an effect on sebum and to evaluate potential direct effects of BoNT/A on sebocytes, we next examined the effect of a commercial preparation of BoNT/A complex (onabotA) in our in vitro model of oily skin. In parallel, to understand if any potential effect of BoNT/A on sebocytes was mediated via cleavage of SNAP-25, or perhaps via a non-classical mechanism involving interaction with cell surface receptors, we also examined the effect of a research grade recombinant version of the BoNT/A binding domain (rH_C_/A) that lacks the SNAP-25 cleaving enzymatic portion of BoNT/A. SEB-1 cells were treated for 24 h with onabotA (100 units in 0.25 mL: ~20 pM) or rH_C_/A (20 or 200 pM) alone or in the presence of OA (0.05 mg/mL). As shown in Figure 3, OA alone significantly increased lipogenesis in SEB-1 cells compared with control (*p* < 0.001). Further, onabotA (*p* < 0.01) or rH_C_/A (*p* < 0.001) alone also increased lipogenesis in SEB-1 cells compared with control. Importantly, co-treatment with either onabotA (*p* < 0.05) or rH_C_/A (*p* < 0.001) reduced the lipogenesis induced by OA. Lipogenesis in sebocytes treated with OA and either onabotA or rH_C_/A was higher than in sebocytes treated with either onabotA or rH_C_/A alone, but lower than in sebocytes treated with OA alone (Figure 3).

### 2.3. Dose-Dependent Effects of BoNT/A on Lipogenesis in SEB-1 Cells

**Figure 4:**
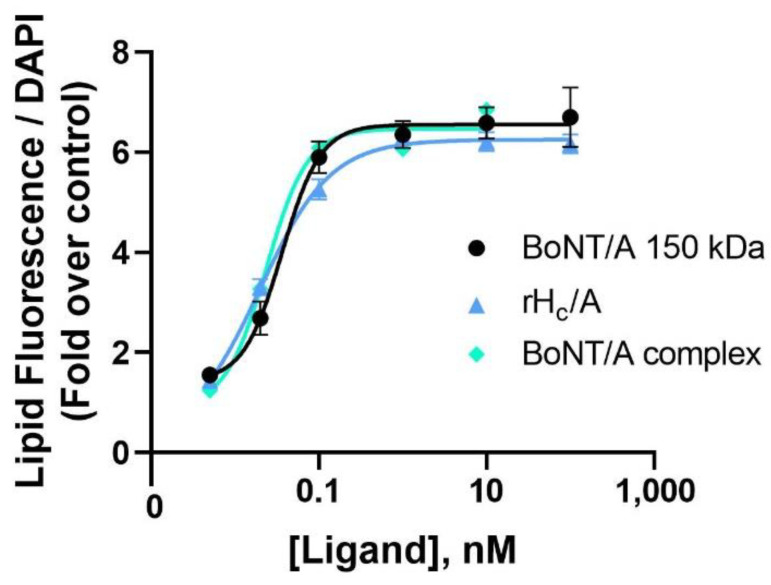
Potency of BoNT/A complex, BoNT/A 150 kDa, and rH_C_/A on naive SEB-1 cell lipogenesis. Dose–response curves showing the amount of intracellular lipids per cell, measured by fluorescence lipid droplet assay and normalized to cell number using DAPI staining, after 24 h treatment with either BoNT/A complex (green, 5 pM–10 nM, EC_50_ = 23 ± 1.58 pM; r^2^ = 0.99), BoNT/A 150 kDa (black, 5 pM-100 nM, EC_50_ = 34 ± 6.31 pM; r^2^ = 0.94), or rH_C_/A (blue, 5 pM-100 nM, EC_50_ = 18 ± 1.81 pM; r^2^ = 0.98). Data shown as fold over control (untreated cells). Data represent means from 5 replicates ± SEM. EC_50_ = half maximal effective concentration.

To further characterize the effect of BoNT/A on lipogenesis, increasing amounts of BoNT/A and BoNT/A derivatives were tested in SEB-1 cells. A range of doses of onabotA (0.1–20 pM) were applied to SEB-1 cells for 24 h; a significant effect of onabotA on lipid accumulation was observed at the 10 and 20 pM doses (Figure S2). Due to the limited amount of toxin in individual vials of onabotA preventing the establishment of a full dose response, we also evaluated the dose–response relationship of research grade native BoNT/A complex, BoNT/A 150 kDa, and BoNT/A binding domain (rH_C_/A). Dose–response curves of BoNT/A complex (EC_50_ = 23 ± 1.58 pM; r^2^ = 0.99), BoNT/A 150 kDa (EC_50_ = 34 ± 6.31 pM; r^2^ = 0.94), and rH_C_/A (EC_50_ = 18 ± 1.81 pM; r^2^ = 0.98) ranging from 0.1–10 µM showed very similar potencies and efficacy on lipid production (Figure 4).

**Figure 5:**
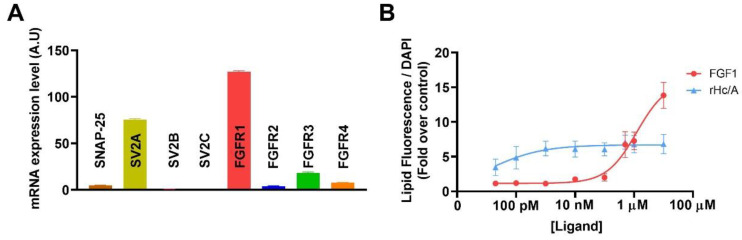
Expression of known and potential BoNT/A receptors and SNARE target in sebocyte (SEB-1) cells and potency of rH_C_/A and fibroblast growth factor 1 (FGF1) on SEB-1 cell lipogenesis. (**A**) RNA-Seq from SEB-1 cells was performed 3 days after plating to evaluate the mRNA expression level of synaptosomal-associated protein 25 (SNAP-25), synaptic vesicle protein 2 A-C (SV2A-C), and FGFR1-4. Data represent means from 5 replicates + SEM. (**B**) Dose–response curves showing the amount of lipids per cell, measured using a Nile red fluorescent dye (lipid droplet fluorescent assay) and normalized to cell number using DAPI staining, after 24 h treatment with FGF1 or rH_C_/A (5 pm–10 µM). Data shown as fold over control (untreated cells). Goodness of fit calculation showed r^2^ values of r^2^ = 0.48 (rH_C_/A) and r^2^ = 0.79 (FGF1). Data represent means from 5 replicates ± SEM.

Since the effect of rH_C_/A, which is devoid of the enzymatic portion of the toxin, on lipogenesis suggested a non-classical effect of BoNT/A on sebocyte lipogenesis, we next tested if the cellular components required for classical BoNT/A activity (i.e., synaptic vesicle protein 2 [SV2], FGFR, SNAP-25) are present in these cells [55]. We thus analyzed gene expression in SEB-1 cells using RNA-Seq (Figure 5A). Unlike neurons, sebocytes have little to no expression of SNAP-25 RNA, the substrate for BoNT/A, although they express RNA for some of its known and potential receptors, including SV2A [54], FGFR1, and FGFR3 [49].

Given the robust expression of FGFR1 in sebocytes, we tested whether natural FGFR ligands (FGFs 1, 2, 4, 10, 18, 19, 21) were able to induce lipogenesis in SEB-1 cells and found that only FGF1 induced significant lipogenesis (Figure S3). Interestingly, FGF1 showed a much lower potency, but higher efficacy, than rH_C_/A. The efficacy of FGF1 was similar to that observed with OA (Figure 3 versus Figure 5B).

### 2.4. FGFRs Are Involved in BoNT/A-Mediated Inhibition of OA-Induced Lipogenesis

**Figure 6:**
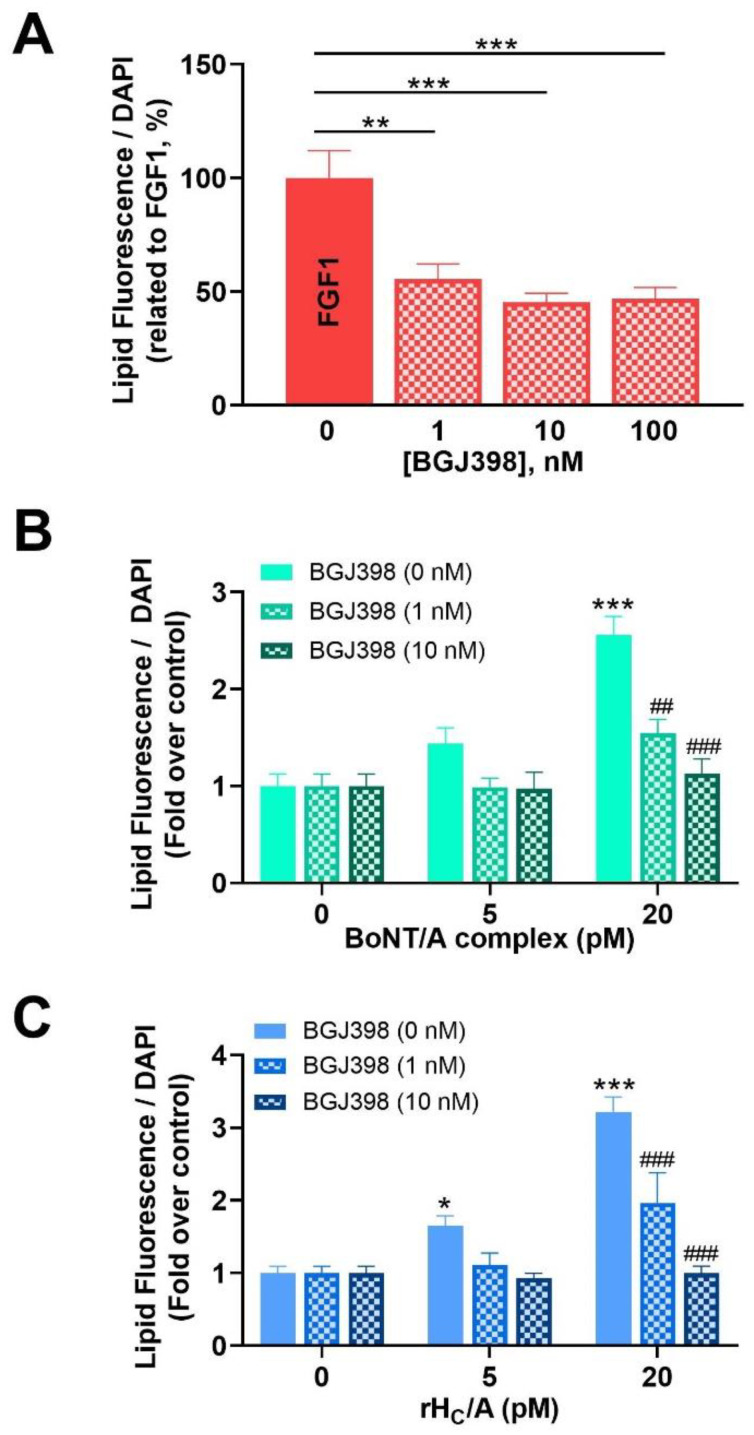
Effect of the FGFR-specific kinase inhibitor BGJ398 on FGF1-, BoNT/A complex-, and rH_C_/A-stimulated sebocyte (SEB-1) cell lipogenesis. (**A**) Dose response of BGJ398 (1–100 nM) on lipid accumulation, as measured by a Nile red fluorescent dye (lipid droplet fluorescence assay) and normalized to cell number using DAPI staining, induced by FGF1 (shown as % of inhibition versus FGF1 alone). Note that 10 nM and 100 nM BGJ398 similarly reduced the FGF1-induced lipid accumulation by ~50%. ** *p* < 0.01, *** *p* < 0.001 vs. FGF1 (0 nM BGJ398). (**B**,**C**) SEB-1 cells were treated with BGJ398 (1, 10 nM) starting 30 min before co-treatment with either BoNT/A complex (B; 5, 20 pM) or rH_C_/A (C; 5, 20 pM) for 24 h. Intracellular lipid accumulation was quantified using the lipid droplet fluorescence assay and normalized to cell number using DAPI staining. Data shown as fold over control (untreated cells). Note the inhibitory effect of BGJ398 on both BoNT/A complex- and rH_C_/A-induced lipogenesis. Data represent means from 5 replicates + SEM. * *p* < 0.05, *** *p* < 0.001 vs. control (0 pM BGJ398); ## *p* < 0.01, ### *p* < 0.001 vs. BoNT/A complex or rH_C_/A alone.

Given the ability of FGF1 to induce lipogenesis, and reports of BoNT/A binding FGFR, the involvement of FGFR in sebocyte lipogenesis was further evaluated. Increasing concentrations of the FGFR1-3 inhibitor BGJ398 [54] (1–100 nM) decreased the FGF1-induced lipogenesis by ~45–55% (*p* < 0.01) (Figure 6A). A dose of either 1 or 10 nM BGJ398 was then used in combination with either BoNT/A complex (Figure 6B) or rH_C_/A (Figure 6C). At a dose of 20 pM of BoNT/A complex or rH_c_/A, BGJ398 decreased lipogenesis induced by BoNT/A complex or rH_C_/A by ~60% (*p* < 0.01) and ~90% (*p* < 0.001), respectively (Figure 6B,C). These results suggest that the effects of BoNT/A on sebocyte lipogenesis are mediated at least partly via FGFR and independent from the light chain activity.

### 2.5. Effect of SV2 and Ganglioside Mutants of rH_C_/A on SEB-1 Lipogenesis

**Figure 7:**
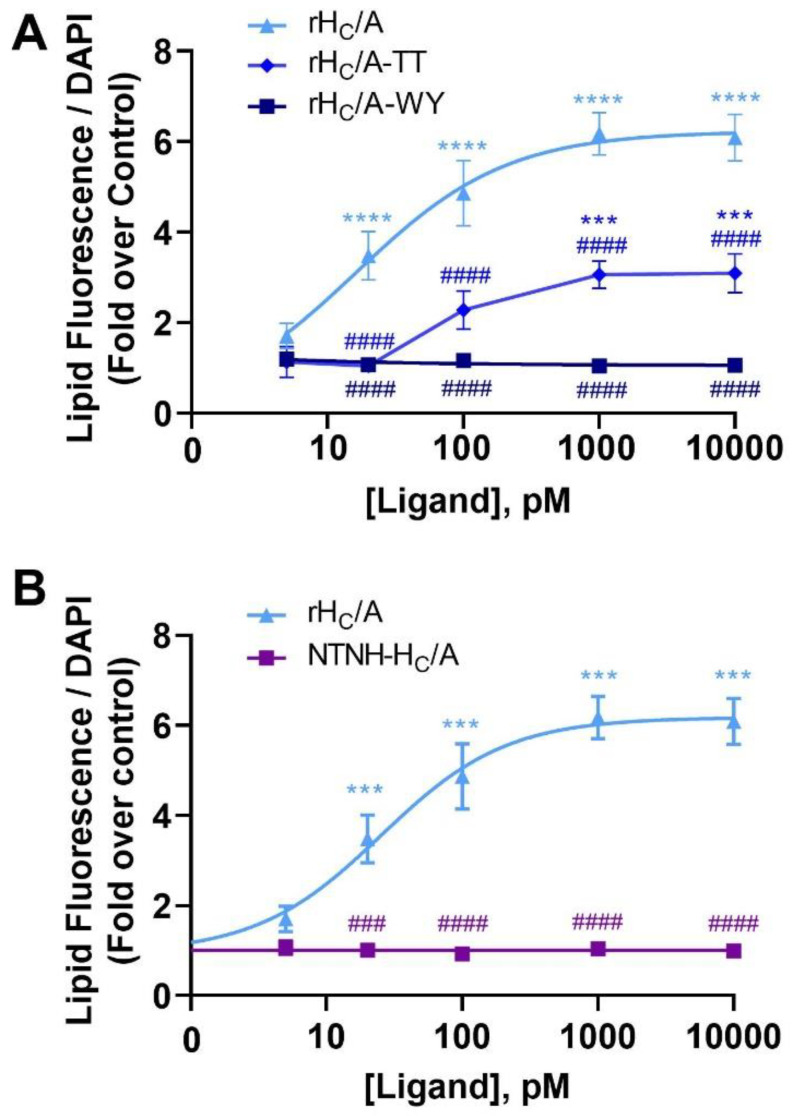
SV2 and ganglioside binding site mutants have decreased or no effects on lipogenesis in SEB-1 cells. (**A**) Dose–response curves showing the amount of lipid per cell, as measured by a Nile red fluorescent dye (lipid droplet fluorescence assay) and normalized to cell number using DAPI staining, after 24 h treatment with either wild-type or mutant recombinant BoNT/A binding domain; rH_C_/A, rH_C_/A-TT, or rH_C_/A-WY (5 pM–10 nM). Data shown as fold over control (untreated cells). Goodness of fit calculation showed r^2^ values of r^2^ = 0.72 (rH_C_/A), r^2^ = 0.90 (rH_C_/A-TT), and r^2^ = 0.04 (rH_C_/A-WY). (**B**) Dose–response curves showing the amount of lipid per cell, as measured by a Nile red fluorescent dye (lipid droplet fluorescence assay) and normalized to cell number using DAPI staining, after 24 h treatment with rH_C_/A or binding domain homolog of nontoxic nonhemagglutinin (NTNH-H_C_/A) (5 pM–10 nM). Data shown as fold over control (untreated cells). Goodness of fit calculation showed r^2^ values of r^2^ = 0.81 (rH_C_/A) and r^2^ = 0.00 (NTNH-H_C_/A). Data are representative of 2 independent experiments showing means from 5 replicates ± SEM. *** *p* < 0.001 vs. control, **** *p* < 0.0001. ### *p* < 0.001, #### *p* < 0.0001 vs. rH_C_/A.

To further characterize the effect of BoNT/A on sebocyte lipogenesis, we tested if SV2 (rH_C_/A-TT) and ganglioside (rH_C_/A-WY) mutant variants of rH_C_/A [56,57,58] could affect the ability of rH_C_/A to induce lipid accumulation. Dose–response curves showed that, compared with wild-type rH_C_/A which induced significant lipogenesis (*p* < 0.001 vs. control), the SV2 mutant variant rH_C_/A-TT had reduced ability (*p* < 0.0001 vs. rH_C_/A) to induce lipogenesis and the ganglioside mutant variant rH_C_/A-WY has completely lost the ability to induce lipogenesis (*p* < 0.0001 vs. rH_C_/A) (Figure 7A). Of interest, the effect of the BoNT/A binding domain is specific to the toxin, as a variant of NTNH, corresponding to the binding domain of NTNH (rNTNH-H_C_/A), was unable to induce lipogenesis (*p* < 0.0001 vs. rH_C_/A) (Figure 7B).

### 2.6. Effect of BoNT/A on OA-Treated Human Primary Sebocytes

**Figure 8:**
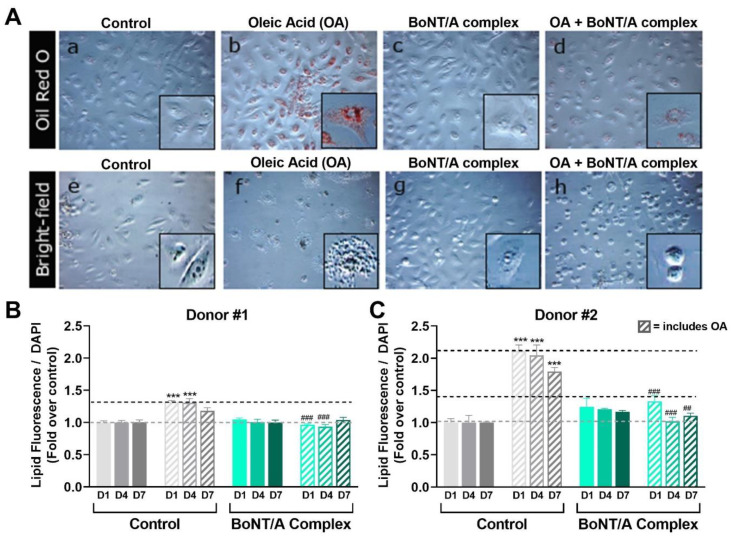
Effect of native BoNT/A complex on naive and OA-stimulated human primary sebocytes. (**A**) Human primary sebocytes (donor #1) were treated with 0.125 mg/mL OA, BoNT/A complex (20 pM), or both (OA + BoNT/A complex). Lipid accumulation was visualized by microscopy after Oil Red O staining (upper panels) and using bright field (lower panels). OA-induced lipid accumulation is visible in panel **b** and holocrine-like secretion is visible in panel **f**. Co-treatment with 20 pM BoNT/A complex for 1 day reduced OA-induced accumulation of intracellular lipids (panel **d**) and prevented or delayed holocrine secretion (panel **h**). Treatment with BoNT/A complex alone did not affect the amount of sebum lipids (panel **c**) nor morphology of the cells (panel **g**). Square boxes show higher magnification images of each condition. (**B**,**C**) Human primary sebocytes (donor #1 and #2) were treated with 0.125 mg/mL OA, and intracellular lipids were measured using a Nile red fluorescent dye (lipid droplet fluorescence assay) and normalized to the cell number using DAPI staining. Data shown as fold over control (untreated cells). Though donors #1 and #2 had different amounts of lipid increases after OA treatment (1, 4, or 7 days), co-treatment with 20 pM BoNT/A complex prevented the OA-induced increase in lipids in both, keeping it at, or close to, the baseline level (100% and 130% on day 1, respectively). Data represent means from 5 replicates + SEM. *** *p* < 0.001 vs. control; ## *p* < 0.01, ### *p* < 0.001 vs. OA.

Knowing the effect of OA and BoNT/A on lipid production in SEB-1 cells, we also evaluated if OA and BoNT/A had similar effects on lipogenesis in human primary sebocytes, which may be more clinically relevant. Human primary sebocytes from 2 different donors were treated with 0.125 mg/mL OA, a dose that causes lipid accumulation and holocrine secretion in SEB-1 cells, and normalized to the baseline amount of lipogenesis for each donor (donor #1: ~4000 RFU; donor #2: ~8000 RFU). As shown in Figure 8A, OA led to visual accumulation of intracellular lipids (panel b) and eventually to holocrine-like secretion, seen as a halo of lipids after cell rupture (panel f). Co-treatment with a clinically relevant concentration of BoNT/A complex (20 pM) for 24 h reduced OA-induced accumulation of intracellular lipids (panel d) and prevented or delayed holocrine secretion (panel h). Treatment with BoNT/A complex in the absence of OA did not affect the amount of sebum lipids (panel c) nor morphology of the primary sebocytes (panel g). This contrasts with what was observed in SEB-1 cells; this is likely due to inherent differences between SEB-1 cells and primary sebocytes, as SEB-1 cells are also much more sensitive to OA. Quantitative analysis of lipid droplet fluorescence in primary sebocytes from both donors shows that OA induced a significant lipid accumulation after 24 h, which was observed through 7 days, although the response was less pronounced in donor #1 than donor #2 (1.3-fold vs. 2.1-fold on day 1). Co-treatment with OA and BoNT/A complex prevents the lipogenesis induced by OA treatment (0.125 mg/mL) as shown in Figure 8B,C. Specifically, co-treatment with 20 pM BoNT/A complex for 1, 4, or 7 days prevented the OA-induced increase in lipids, keeping it at, or close to, the baseline level (100% and 127% on day 1, respectively; *p* < 0.01 vs. OA alone). Quantitative analysis also showed that treatment with BoNT/A complex in the absence of OA had no significant effect in primary sebocytes from either donor.

## 3. Discussion

Clinical observations suggest that commercial preparations of BoNT/A have benefits beyond those mediated by its classical muscle relaxation effects. One such benefit is improvement in skin quality via reduction of excessive skin oiliness, or seborrhea [39,40,41,42,43,44]. Here, we provide evidence to suggest that BoNT/A directly affects sebocyte lipogenesis independent of its enzymatic activity using an in vitro model for seborrhea.

We developed an in vitro model for seborrhea using SEB-1 sebocyte cells treated with oleic acid (OA), a robust and efficacious stimulator of SEB-1 lipogenesis. However, little is currently known about the cellular mechanism of action of OA. In our model, both research grade BoNT/A complex and a commercial preparation of BoNT/A (onabotA) inhibited OA-induced lipogenesis, consistent with clinical reports of BoNT/A reducing seborrhea [39,40,41,42,43,44]. We also observed this inhibitory effect with the recombinantly expressed wild-type BoNT/A binding domain (rH_C_/A), demonstrating that the effect was independent of the enzymatic activity of BoNT/A. This is consistent with our RNA-Seq analyses, which showed little to no expression of SNAP-25 RNA in sebocytes, although future studies examining protein expression of SNAP-25 in sebocytes are needed. Surprisingly, given clinical reports of BoNT/A reducing seborrhea [39,40,41,42,43,44], treatment of unstimulated SEB-1 cells with BoNT/A increased lipogenesis. Research grade native BoNT/A complex, BoNT/A 150 kDa, and rH_C_/A also induced lipogenesis in SEB-1 cells with similar (pM) potency and efficacy. The low amount of toxin in commercial preparations of BoNT/A did not allow us to establish a full dose–response and calculate an EC_50_ value for onabotA. These data suggest that the effect of BoNT/A is modulatory rather than strictly inhibitory, increasing or decreasing lipogenesis depending on the state of the sebocyte cell.

To perhaps better model the clinical situation, we also evaluated the effect of OA and BoNT/A on primary sebocytes from 2 human female donors. The primary sebocytes were much less sensitive to OA than the male donor-derived immortalized sebocyte (SEB-1) cells; specifically, we saw that the increase in lipogenesis in the primary sebocytes in response to OA was ≥10 times lower than in the SEB-1 cells. It is possible that male-derived sebocytes are more sensitive to lipogenic stimuli due to higher expression of factors and receptors mediating androgen-dependent signaling, such as DHT, androgen receptor (AR), IGF-1, and/or insulin-like growth factor 1 receptor (IGF1R). Androgen-dependent signaling is a key driver of sebocyte lipogenesis and has also been reported to crosstalk with FGFRs [51,52]. The baseline level of lipogenesis (donor #1: ~4000 RFU; donor #2: ~8000 RFU) and the magnitude of lipogenic response upon stimulation with OA also varied between the donors’ cells, further suggesting differences based on the patient/donor. The sebocytes from donor #1, who was receiving PREMARIN estrogen tablets for treatment of menopause, were more responsive to OA than donor #2 with high BMI; hormone status and BMI have both been reported to affect lipogenesis. Notably, the primary sebocytes did not respond to BoNT/A alone. However, as with what was observed in SEB-1 cells and consistent with clinical reports of BoNT/A reducing seborrhea, BoNT/A reduced the lipogenic effect of OA in these primary cells back to baseline levels. The reason for the difference between effect of BoNT/A in SEB-1 and primary sebocytes is unknown and warrants further study, including evaluation of additional primary sebocytes and sebocyte cell lines.

We also tested the effects of mutant variants of the BoNT/A binding domain, either lacking binding to gangliosides (rH_C_/A-WY [49,50]) or with reduced binding to SV2 (rH_C_/A-TT [49,51]), which had no or reduced effect on SEB-1 lipogenesis, respectively. These results suggest the effect of BoNT/A on sebocyte lipogenesis is, at least partly, dependent on its classical receptors, or alternatively that these mutations alter the structure of the binding domain. A recombinant binding domain homolog variant of NTNH (rNTNH-H_C_/A), which is structurally similar to the binding domain of BoNT/A, but lacks receptor binding sites, had no effect on SEB-1 lipogenesis, further suggesting that the effect of BoNT/A is receptor mediated.

FGFRs and FGFs have previously been reported to affect sebocyte lipogenesis [45,46]. This is consistent with our RNA-Seq analyses showing that sebocytes express mRNA transcripts for FGFRs, with robust levels of FGFR1 and moderate expression of FGFR3. Interestingly, in our SEB-1 model, among the selected FGFs evaluated (i.e., FGFs 1, 2, 4, 10, 18, 19, 21), only FGF1 affected lipogenesis, suggesting FGF1 is unique in its ability to alter lipogenesis. The lack of effect of other FGFs could be due to the selected 24 h time point, precluding observation of FGF-mediated effects on proliferation at later time points, and/or non-optimal dosing. Indeed, FGF2 has been shown to promote sebocyte proliferation at the expense of sebocyte differentiation, thereby reducing sebocyte lipogenesis [45,46]. The lipogenic effect of FGF1 in SEB-1 cells was blocked by the FGFR-specific inhibitor BGJ398, suggesting the effect of FGF1 on lipogenesis is via FGFR.

We have previously reported that the binding domain of BoNT/A (rH_C_/A) binds to FGFRs and acts like an FGFR ligand [49,55]. Consistent with these previous observations, we show here that the lipogenic effect of BoNT/A and rH_C_/A on SEB-1 cells was blocked by the FGFR-specific inhibitor BGJ398, suggesting the effect of BoNT/A on lipogenesis is via FGFR. Interestingly, when comparing the potency and efficacy of FGF1 and BoNT/A, BoNT/A is more potent (pM vs. nM) but less efficacious. Indeed, the maximal amount of lipogenesis observed after treatment with BoNT/A is less than what is observed after treatment with FGF1 (or OA). It is unclear why BoNT/A and FGF1, both acting via FGFR, have different potency and efficacy. A possibility is that BoNT/A has higher potency due to interactions with additional sebocyte (co) receptors, including gangliosides and SV2. Mutating BoNT/A residues, interfering with ganglioside or SV2 binding, has been shown to disrupt rH_C_/A-induced FGFR dimerization on neuronal cells [59]. Here, we observe that the same mutations disrupt BoNT/A-induced lipogenesis, suggesting these residues either affect binding to FGFR and/or that interactions with gangliosides and SV2 are required for BoNT/A to interact with FGFR. It should also be kept in mind that the sebocyte media contain FGFs, preventing evaluation of lipogenesis in the absence of FGF. The observed lower efficacy could be due to BoNT/A acting as a partial or biased agonist. Partial agonism could also help explain why BoNT/A inhibits lipogenesis in OA-stimulated SEB-1 sebocytes.

Future studies to help further elucidate the mechanism of action of BoNT/A-mediated reductions in sebum levels are warranted and effects of other Botulinum neurotoxins should be evaluated. Additional research is also needed to understand the clinical translatability of our model, and to further explore the effect of FGF1 and the role of FGFR in sebocyte lipogenesis, including the potential of using kinase inhibitors for reducing skin oiliness.

Overall, the data presented herein show that BoNT/A can modulate sebocyte lipogenesis and support the hypothesis that the observed clinical effects of BoNT/A on sebum could be due, at least in part, to a direct effect of BoNT/A on sebocytes. The data also raise the question as to whether the clinical effect of BoNT/A is always to reduce sebum or if the effect may depend on the baseline level of sebum, perhaps reducing sebum in subjects with seborrhea and either not affecting or increasing sebum in normal and dry skin subjects.

## 4. Materials and Methods

### 4.1. Primary Cells and Cell Lines

The human sebocyte cell line SEB-1 was a generous gift from Dr. Thiboutot [47] and human primary sebocytes were purchased from ZenBio (Durham, NC, USA; Table 1). Both cell line and primary cells were cultured in Sebocyte Growth Medium supplied with EGF and bovine pituitary extract (ZenBio, Durham, NC, USA; Cat#: SEB-1).

### 4.2. Drugs and Treatments

**Figure 9:**
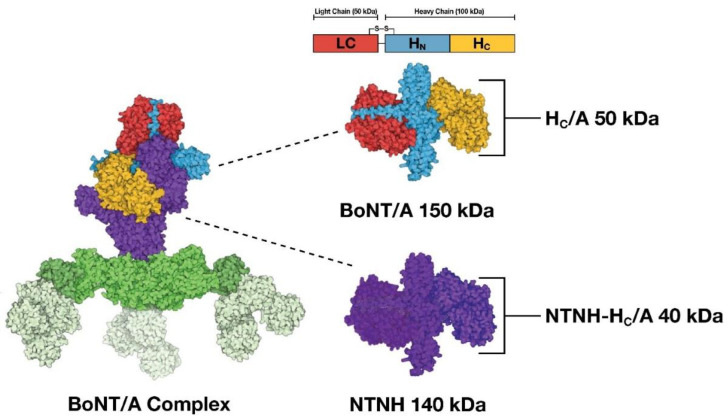
BoNT/A complex and derivatives. BoNT/A complex consists of a 150 kDa Botulinum Neurotoxin Type A (BoNT/A) protein comprised of a light chain (LC; red), and a heavy chain (HC) containing a receptor binding domain (H_C_; yellow) and a translocation domain (H_N;_ blue). This protein complexes with the 140 kDa nontoxic-nonhemagglutinin (NTNH; purple) and hemagglutinins (HAs; green). Adapted by permission from Springer Nature Switzerland AG Customer Service Centre GmbH: Springer Nature; Dong, M., Stenmark, P. The Structure and Classification of Botulinum Toxins. In: Whitcup, S.M., Hallett, M. (eds) Botulinum Toxin Therapy. Handbook of Experimental Pharmacology, vol 263. Copyright © 2019, Springer Nature Switzerland AG [33].

Multiple stimuli and BoNT/A variants were used to examine lipogenesis in SEB-1 cells or human primary sebocytes. SEB-1 cells were selected for these studies over other available sebocyte cell lines, such as SZ95, due to their increased responsiveness to OA-induce lipid stimulation in pilot studies (~20-fold increase in lipids in SEB-1 cells compared to ~2-fold increase in SZ95 cells). SEB-1 cells were treated with the following lipogenesis stimulators for 1 day: OA (0.05 mg/mL; Sigma-Aldrich, St. Louis, MO, USA: Cat# O1383-1G), DHT (100 µM; Sigma-Aldrich, St. Louis, MO, USA: Cat# D-073-1ML), FGFs (FGFs 1, 2, 4, 10, 18, 19, 21; 0.1-0.5 µM; R&D Systems, Minneapolis, MN, USA), rosiglitazone (100 µM; Sigma-Aldrich, St. Louis, MO, USA: Cat# R2408-50MG), CaCl_2_ (2 mM; VWR, Radnor, PA, USA: Cat# 97062-820), ACh (300 µM; Sigma-Aldrich, St. Louis, MO, USA: Cat# A2661-25G), α-MSH (5 µM; Abcam, Cambridge, United Kingdom: Cat# ab120189), or BoNT/A (native research grade BoNT/A complex [Metabiologics, Madison, WI, USA] or onabotulinumtoxinA [Allergan Aesthetics, an AbbVie company, Irvine, CA, USA]) and derivatives (described in Table 2; visualized in Figure 9). BoNT/A and derivatives were diluted in pre-warmed media and serial dilutions were conducted by adding pre-warmed media to obtain the desired dilution. In the case of cotreatment with OA or BGJ398, BoNT/A and derivatives were diluted in pre-warmed media containing OA or BGJ398. Once BoNT/A or derivatives were added to the media, compounds were mixed by up and down pipetting at least 15 times. Cells were then treated by aspiring the entire media and replacing it with media containing the tested compounds. Human primary sebocytes or SEB-1 cells were treated with OA for 1–7 days with or without BoNT/A derivatives (Table 2; Figure 9).

### 4.3. Oil Red O Staining and Bright Field Imaging

For Oil Red O Staining and bright field imaging, cells were seeded into flat bottom 96-well microplates (Genesee Scientific Corp; El Cajon, CA, USA: Cat#: 22-71) at a confluency of 0.08 × 10^6^ cells/cm^2^. After fixation with formalin (10%, 30 min), cells were washed with double-distilled water (ddH2O) twice, followed by 5 min incubation with 60% isopropanol. Then, Oil Red O staining solution (Sigma-Aldrich, St. Louis, MO, USA: Cat# MAK194) was applied for 20 min. After washing 5 times with water, hematoxylin was added. The excess hematoxylin was removed by another 5 washes with ddH2O and the cells processed for microscopy. The Oil Red O solution stained lipid droplets green (Figure 3A) and hematoxylin stained the nuclei blue. Bright field images were taken to assess sebocyte morphology using Incucyte ZOOM (Essen BioScience, Ann Arbor, MI, USA) at 10× magnification.

### 4.4. Lipid Droplets Fluorescence Assay

Intracellular lipid content was measured using the lipid droplets fluorescence assay (Nile Red assay, Cayman Chemical, Ann Arbor, MI, USA: Cat# 500001) following manufacturer’s protocol. Briefly, cells were seeded into flat bottom 96-well microplates (Genesee Scientific Corp; El Cajon, CA, USA: Cat# 22-71) at a confluency of 10,000 cells/well (SEB-1 and primary sebocytes). The next day, cells were incubated for 1–7 days with media only (control) or media plus OA (stimulation) and/or test article (e.g., BoNT/A or rHc/A derivatives). For inhibitor studies, cells were treated with the selective FGFR1-3 inhibitor BGJ398 (1–100 nM) [54] starting 30 min before incubation with media, media plus OA, and/or test articles. Concentration ranges were selected based on existing literature and pilot studies. Next, after fixation and 1 wash with assay buffer, cells were incubated with Nile Red staining solution (which becomes fluorescent in a hydrophobic environment, i.e., lipid droplets) for 30 min, followed by 5–10 min of incubation with DAPI nuclear staining solution (1:1000, MiliporeSigma, Burlington, MA, USA: Cat# D9542). After 1 wash with assay buffer, the fluorescence intensity of lipid droplets was measured with the fluorescence microscope Nikon Eclipse Ti (Melville, NY, USA) or with an EnVision 2102 Multilabel plate reader to detect fluorescein isothiocyanate (FITC) (Ex/Em 485/535nm) and DAPI (Ex/Em 358/461).

### 4.5. RNA Sequencing

SEB-1 cells were seeded in 6-well plates at the density of 1 × 10^6^ cells/well. After 3 days, media was removed, and cells were lysed in 500 µL of RLT buffer (Qiagen, Hilden, Germany: Cat#: 79216). RNA was extracted using RNeasy Qiagen mini kit (Qiagen, Hilden, Germany: Cat# 74104) following instructions from manufacturer. Libraries for RNA-Seq were prepared with KAPA Stranded RNA-Seq Kit (Roche, Basel, Switzerland: Cat# 07962282001). The workflow consists of mRNA enrichment, cDNA generation, and end repair to generate blunt ends, A-tailing, adaptor ligation, and PCR amplification. Different adaptors were used for multiplexing samples in one lane. Sequencing was performed on Illumina HiSeq 3000 for a single read 50 run. Data quality check was done on Illumina SAV. Demultiplexing was performed with Bcl2fastq2 v 2.17 conversion software (Illumina, San Diego, CA, USA) program. The reads were first mapped to the latest University of California Santa Cruz (UCSC) transcript set (https://genome.ucsc.edu; accessed on 1 June 2019) using Bowtie2 version 2.1.0 [60] and gene expression levels were estimated using RSEM (RNA-Seq by Expectation-Maximization) software v1.2.15 [61]. TMM (trimmed mean of M-values) was used to normalize the gene expression.

### 4.6. Statistical Analyses

Statistical analyses were performed using GraphPad Prism (versions 8.4 and 9.1) software (GraphPad Software, San Diego, CA, USA). Values are mean ± standard error of the mean (SEM). Significant differences between treatment groups were determined either by one-way analysis of variance (ANOVA) with post hoc analysis using Student–Newman–Keuls multiple comparison test or two-way analysis of variance with post hoc analysis using Bonferroni multiple comparison test. Values of *p* < 0.05 were accepted as significant. Dose–response curves were assessed using sigmoidal 4-parameter logistic model applying curve with no constraint. Where appropriate, the goodness of the curve fit (r^2^) was calculated and is reported in associated figure legends.

## Figures and Tables

**Table 1:** Sebocyte cell line information.

Cell Type	Description	Source
Human primary sebocyte donor #1	Sebocytes were isolated from the facial skin of a Caucasian 66-year-old female donor under PREMARIN^®^ (conjugated estrogen tablets, USP) treatment for menopause	ZenBio^®^ (Durham, NC, USA) Cat# SEBM011514B
Human primary sebocyte donor #2	Sebocytes were isolated from the facial skin of a Caucasian 59-year-old female overweight (body mass index: 27.6 kg/m^2^) donor	ZenBio^®^ (Durham, NC, USA) Cat# SEBM022614B
SEB-1 immortalized sebocyte cell line	Sebocytes were isolated from the facial skin of a 55-year-old male donor and SV40 immortalized [47]	Dr. Diane Thiboutot

**Table 2:** BoNT/A test materials.

Name	Description	Reference
onabotA (BOTOX^®^, Allergan Aesthetics, an AbbVie company, Irvine, CA, USA)	Commercially available preparation of BoNT/A	
BoNT/A complex (Metabiologics, Madison, WI, USA)	Research grade botulinum neurotoxin type A, native complex; purified from *Clostridia*	
BoNT/A 150 kDa (Metabiologics, Madison, WI, USA)	Research grade botulinum neurotoxin type A, holotoxin, 150 kDa	
rH_C_/A	Recombinant binding domain of BoNT/A	[56]
rH_C_/A-WY	Recombinant binding domain of BoNT/A with mutations W1266L and Y1267S that prevent binding to gangliosides (sugar receptors for BoNT/A)	[56,57]
rH_C_/A-TT	Recombinant binding domain of BoNT/A with mutations T1145A and T1146A that reduces binding to synaptic vesicle protein 2 (SV2), a protein receptor for BoNT/A	[56,58]
rNTNH-H_C_/A	Recombinant binding domain homolog of nontoxic nonhemagglutinin (NTNH)	

## Data Availability

Data reported in this manuscript may be requested by contacting AbbVie Inc.

## Supplementary Materials

The following supporting information can be downloaded at: https://www.mdpi.com/article/10.3390/toxins14100708/s1, Figure S1: Effect of different lipid droplet inducers on cultured SEB-1 cells.; Figure S2: Potency of onabotA on naive SEB-1 cell lipogenesis; Figure S3: Effects of FGFs on lipogenesis.

## Abbreviations

BoNT/A	botulinum neurotoxin type A
OnabotA	onabotulinumtoxinA
α-MSH	alpha melanocyte-stimulating hormone
EGF	epidermal growth factor
FGF	fibroblast growth factor
FGFR	fibroblast growth factor receptors
IGF-1	insulin-like growth factor 1
IGF1R	insulin-like growth factor 1 receptor
ACh	acetylcholine
LC	light chain of BoNT/A
HC	heavy chain of BoNT/A
H_C_	receptor binding domain
rH_C_/A	recombinant receptor binding domain of BoNT/A
H_N_	translocation domain
NTNH	nontoxic nonhemagglutinin protein
rNTNH-H_C_/A	recombinant binding domain homolog of nontoxic nonhemagglutinin protein
HAs	hemagglutinins
SNAP-25	synaptosomal-associated protein-25 kDa
DHT	dihydrotestosterone
CaCl_2_	calcium chloride
PPAR	peroxisome proliferator-activated receptor
TGF-α	transforming growth factor alpha
OA	oleic acid
EC_50_	half maximal effective concentration
SV2	synaptic vesicle protein 2
AR	androgen receptor
