# Supplementary material for: Botulinum Neurotoxin Type A Directly Affects Sebocytes and Modulates Oleic Acid-Induced Lipogenesis
Source: Toxins (Basel). 2022 Oct 15;14(10):708. doi: 10.3390/toxins14100708 (PMC9609209; doi:10.3390/toxins14100708)
Supplement: Supplementary file 1 [file toxins-14-00708-s001.zip › toxins-1896464-supplementary.pdf]

toxins

Supplementary Materials: Botulinum Neurotoxin Type A Directly Affects Sebocytes and Modulates Oleic Acid-Induced Lipogenesis

Karen Brami-Cherrier, Alex Chernavsky, Hui You, Sergei A. Grando, Amy Brideau-Andersen and Birgitte Sondergaard

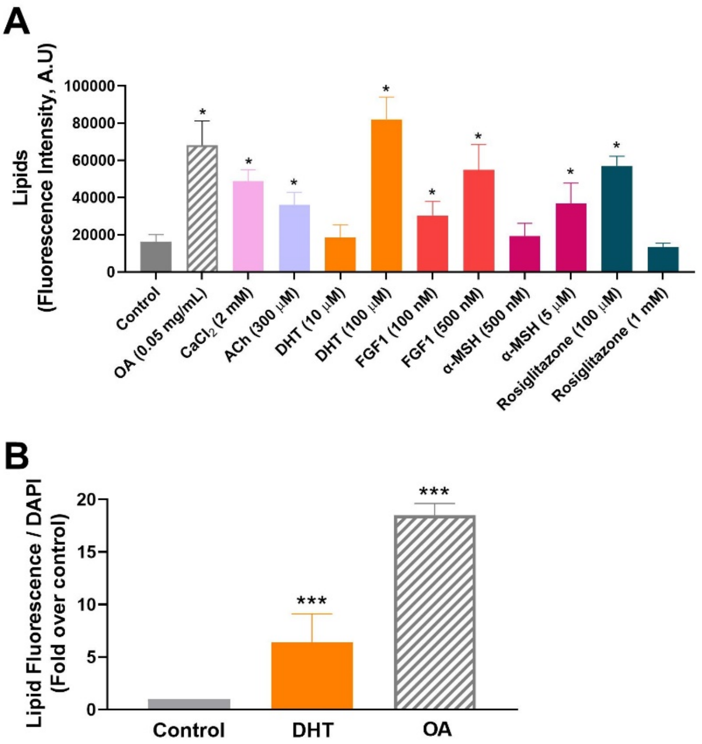

Figure S1. Effect of different lipid droplet inducers on cultured SEB-1 cells. (**A**) SEB-1 cells were treated for 24 hours with oleic acid (OA) (0.05 mg/mL), dihydrotestosterone (DHT) (100 µM), calcium chloride ($CaCl_2$) (2 mM), acetylcholine (ACh) (300 µM), fibroblast growth factor 1 (FGF1) (100 nM or 500 nM), α-melanocyte-stimulating hormone (α-MSH) (500 nM or 5 µM), or rosiglitazone (100 µM or 1 mM); intracellular lipids were measured using a lipid droplets fluorescence assay. (**B**) SEB-1 cells treated for 24 hours with DHT (100 µM) and OA (0.05 mg/mL). Accumulated lipids were normalized to cell number based on DAPI and shown as fold over control (untreated cells). Data represent mean + SEM. * $p < 0.05$, *** $p < 0.001$ vs. control.

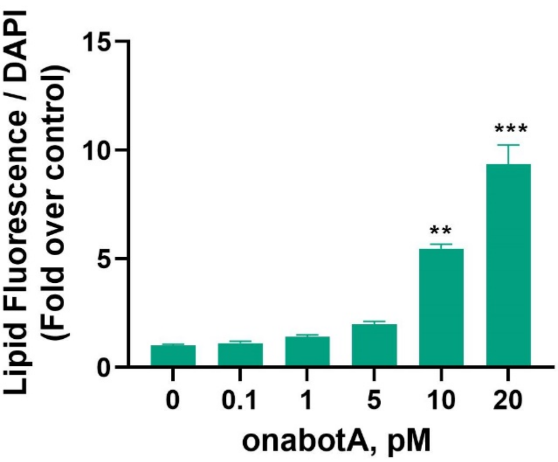

Figure S2. Potency of onabotA on naive SEB-1 cell lipogenesis. Dose response of onabotA (0.1–20 pM) showing the amount of intracellular lipids measured using a Nile red fluorescent dye (lipid droplet fluorescence assay) and normalized to the cell number using DAPI staining. Data shown as fold over control (untreated cells). There was a significant effect of onabotA on intracellular lipid accumulation starting at 10 pM. Data represent means from 5 replicates + SEM. ** $p < 0.01$, *** $p < 0.001$ vs. untreated control.

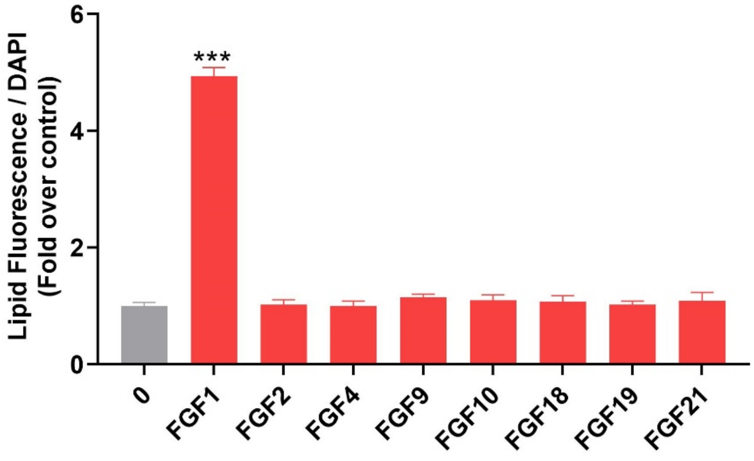

Figure S3. Effects of FGFs on lipogenesis. Bar graph representing the lipids/cell increase after treatment with FGF1, FGF2, FGF4, FGF9, FGF10, FGF18, FGF19 or FGF21 (0.5 μM). Note that only FGF1 was able to induce a significant increase in lipid production. Data represent mean + SEM. *** $p < 0.001$ vs. control (untreated cells).
